# Research Progress on F-P Interference—Based Fiber-Optic Sensors

**DOI:** 10.3390/s16091424

**Published:** 2016-09-03

**Authors:** Yi Wen Huang, Jin Tao, Xu Guang Huang

**Affiliations:** 1 Guangzhou Key Laboratory for Special Fiber Photonic Devices and Applications, School of Information and Optoelectronic Science and Engineering, South China Normal University, Guangzhou 510006, China; 20133203051@m.scnu.edu.cn; 2 State Key Laboratory of Optical Communication Technologies and Networks, Wuhan Research Institute of Posts Telecommunications, Wuhan 430074, China; taojin@wri.com.cn; 3 Specially Functional Fiber Engineering Technology Research Center of Guangdong Higher Education Institutes, Guangdong Provincial Engineering Technology Research Center for Microstructured Functional Fibers and Devices, South China Normal University, Guangzhou 510006, China

**Keywords:** interferometric fiber optic sensor, F-P cavity, fringe contrast, wavelength shift

## Abstract

We review our works on Fabry-Perot (F-P) interferometric fiber-optic sensors with various applications. We give a general model of F-P interferometric optical fiber sensors including diffraction loss caused by the beam divergence and the Gouy phase shift. Based on different structures of an F-P cavity formed on the end of a single-mode fiber, the F-P interferometric optical sensor has been extended to measurements of the refractive index (RI) of liquids and solids, temperature as well as small displacement. The RI of liquids and solids can be obtained by monitoring the fringe contrast related to Fresnel reflections, while the ambient temperature and small displacement can be obtained by monitoring the wavelength shift of the interference fringes. The F-P interferometric fiber-optic sensors can be used for many scientific and technological applications.

## 1. Introduction

The Fabry-Perot (F-P) interferometric optical fiber sensor is one of the most widespread types of fiber-optic sensors, due to its properties of being versatile, simple, responsive, precise and immune to environmental noise [1,2,3]. They have been widely applied in various fields, such as biochemical sensing, seismic and sonar, smart structure monitoring, the oil industry and the aerospace industry [1].

Precise monitoring of temperature, refractive index (RI) and displacement is of great importance for modern scientific and technological applications. There are many other methods for the measurements of these parameters. A time-to-digital-converter–based complementary metal oxide semiconductor (CMOS) smart sensor was proposed for temperature measurement by detecting a pulse with a width proportional to the measured temperature. The effective resolution of the sensor was demonstrated to be better than 0.16 °C [4]. A wireless system with silicon carbide wireless electronics was proposed for the measurement of extreme temperature. This system can achieve a measurement of temperature up to 450 °C [5]. Consisting of a two-core fiber, an optic sensor for high-performance measurements of the RI of liquids was proposed with the largest sensitivity of 3119 nm per RI unit for the RI ranging from 1.3160 to 1.3943 [6]. With a semi-cylindrical-shaped metal layer deposited on a tapered optical fiber, a surface plasmon resonance optical fiber sensor can achieve the measurement of the RI of metal, by monitoring the first three resonance peaks in the transmission spectrum formed by different hybrid surface plasmon modes [7]. A sensor consisting of a laser interferometer, a wavelength encoder and an electronic computing device as well as a displacement indicator was proposed for RI sensing based on the principle of fringe-counting interferometers [8]. With a theoretical resolution better than 0.417 nm, a small displacement-sensing system based on multiple total internal reflections in heterodyne interferometry was proposed [9]. The moiré fringe multiplication technique can be also used to measure small displacements [10]. The interferometric measurement of distance using a femtosecond frequency comb was demonstrated. When the optimal spectral width was used, a fringe contrast higher than 90% could be obtained for kilometer distances in air [11]. By processing techniques and using standard image capture, a technique based on the in-plane fringe enhancement in holographic interferometry generated by two-beam illumination was proposed for the measurements of in-plane displacements. In this technique, the fringes of a moiré pattern formed between two interferograms indicate in-plane displacements [12]. Based on multimode interference and reimaging theory, a fiber-optic sensor composed of a section of multimode fiber fusion spliced to a single-mode fiber for displacement measurements was proposed [13].

However, those methods suffer from limited resolution, long dynamic sensing times, complex structures, or high cost, which create limitations and bottlenecks for their applications.

In 2008, a technology based on a hybrid structured F-P interferometer, which consists of two F-P cavities formed by fusion splicing a short hollow-core fiber and a piece of single-mode fiber at a photonic crystal fiber, was proposed for monitoring high temperature [14]. A fiber-optic temperature sensor based on a rare-earth activated F-P optical cavity and two other microstructured optical fibers were demonstrated for temperature measurement with a simulated sensitivity of *S* = 315.1 μW/°C [15]. Based on a miniature F-P cavity formed between the air-fiber boundary and a reflective in-fiber metallic splice, a fiber-optic temperature sensor was presented for the measurement of high-temperature up to 1100 °C. It can achieve a stability of ∼10 °C over a period exceeding 300 h at 1100 °C [16].

On the other hand, a temperature-independent F-P RI sensor based on a sealed F-P cavity near the tip of a single-mode fiber was demonstrated. The proposed sensor can offer a RI resolution of ~4 × 10^−5^ [17]. Recently, based on an air microcavity, a fiber-intrinsic F-P interferometer was demonstrated for RI sensing [18]. The air microcavity was formed at a segment of a hollow-core photonic crystal fiber with silica walls. Based on an etching-induced micro air gap near the tip of a single-mode fiber, Ma et al. proposed a simple fiber tip sensor for RI measurement, which achieved a high RI resolution of up to 3.4 × 10^−5^ [19].

An interferometric method can be used to measure the RI of a glass precisely [20,21]. Interferometric techniques with an F-P cavity have been also used for measuring the RI of transparent solids [22,23]. In 2009, a variable-path interferometric method with a prism pair for measuring the RI of optical glasses was demonstrated based on two interferometers. The method can achieve the combined standard uncertainty of 1.6 × 10^−6^ [24]. Those methods mentioned above are non-fiber techniques.

As for the monitoring of small displacement, Liu proposed a novel frequency multiplexing method for addressing low-finesse-fiber F-P sensors by using a white light source and a charge-coupled device (CCD)-based monochromator. The method for displacement measurements can achieve an accuracy of better than 0.01 mm [25]. John reported an F-P metrology for a displacement of up to 50 mm in 2005. The absolute uncertainty of the system was demonstrated to be below 10  pm, which for the largest displacement measured corresponded to a relative uncertainty of 4 × 10^−10^ [26]. In 2009, a displacement metrology and control system based on a dual F-P cavity and an optical frequency comb generator was proposed [27]. The absolute uncertainty of this system was approximately 190 pm for the displacement of 14 μm, while the accurate displacement control was demonstrated with a resolution of approximately 24 pm.

Besides, previous investigations of F-P interferometric optical fiber sensors indicate that they have very broad applications from magnetic fields [28,29,30,31,32], stress/strain [33,34,35,36], vibration [37,38], humidity [39,40,41] and current [42] to gas [43] and pressure [44,45,46,47].

In this article, we firstly give a general model for the basic operation principle of F-P interferometric optical fiber sensors by including the analysis of the Gouy effect and the beam divergence in an F-P cavity. We review our research progress in F-P interferometric optical fiber sensors regarding their sensor designs and applications. F-P interferometric optical fiber sensors for temperature, the RI of liquids and solids, and small displacement measurements are included. Finally, we conclude with an outlook for the potential applications of F-P interferometric optical fiber sensors.

## 2. The Basic Principle of Operation

### 2.1. The Principle

**Figure 1:**
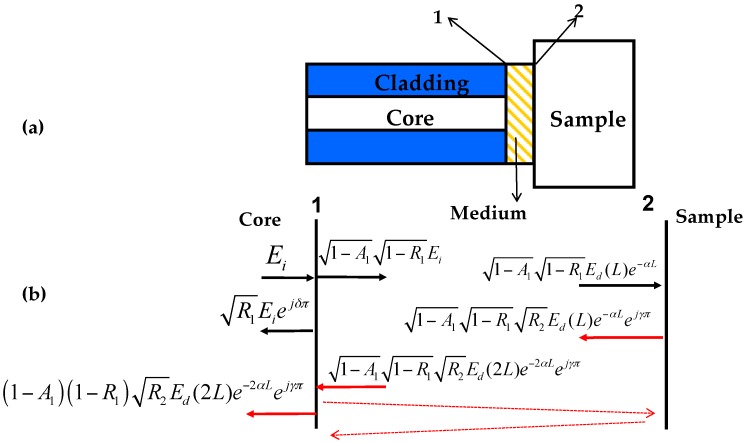
(**a**) Basic structure of the sensing head; (**b**) The field amplitudes at the two reflection surfaces.

The basic structure of a sensing head based on a fiber end is shown schematically in Figure 1a. One can see that the sensing head consists of two reflection surfaces, which are labeled as “1” and “2”, respectively.

They form a short F-P cavity with the length of *L*. The RI of the sample, medium and fiber are denoted as *n_x_*, *n_m_* and *n_f_*, respectively. The power reflectivity on surfaces 1 and 2 is respectively denoted as *R*_1_ and *R*_2_. As the reflection of the incident light on both surfaces is a type of Fresnel reflection, *R*_1_ and *R*_2_ can be obtained from the Fresnel equation [48]. The electric fields on reflection surfaces 1 and 2 are shown in Figure 1b. However, due to high losses and low reflection coefficients, the total contribution from high-order reflections is very small (less than 0.1%), and thus can be neglected (as pictorially illustrated with red dashed lines shown in Figure 1b). Therefore, the system can be treated as a double-beam interferometer, and the total electric field of *E_r_*, which is reflected from two surfaces, can be obtained as follows:

(1) $$E_{r}\;\approx \;\sqrt{R_{1}}E_{i}e^{j\delta \pi }\;+\;\left(1\;-\;A_{1}\right)(1\;-\;R_{1})\sqrt{R_{2}}e^{-2\alpha L}E_{d}(2L)e^{j\gamma \pi }$$

where *E_i_* is the input field from the end of the fiber, and *E_d_*(2*L*) is the diffracted field after a round-trip within the cavity. *A*_1_ is the transmission loss factor on surface 1, which results from the surface imperfections (e.g., roughness); *α* is the absorption coefficient of the optical medium in the cavity; *η* is the coupling efficiency of the reflected optical field being coupled back into the fiber (mode) on surface 2; *δ* and *γ* are, respectively, 1 or 0 as determined by the half-wave loss or none on surface 1 or 2. Due to transverse modal fields which can be approximated as a Gaussian distribution in a single-mode fiber, the normalized optical field *E_i_* and the diffracted field *E_d_* can be obtained as follows [49]:

(2) $$E_{i}(r)\;=\;A/\omega_{0}\exp\left(\;-\;r^{2}/{\omega_{0}}^{2}\right)\;E_{d}(r,z)\;=\;A/\omega (z)\exp\left[\;-\;r^{2}/\omega^{2}(z)\right]\exp\left[\;-\;j\phi (r,z)\right]$$

where $\omega_{0}$, $\omega (z)\;=\;\omega_{0}{\left(1\;+\;z^{2}/f^{2}\right)}^{1/2}$ and $\phi (r,z)\;=\;kz\;+\;kr^{2}/[2z(1\;+\;f^{2}/z^{2})]\;-\;\varphi (z)\;\approx \;kz\;-\;\varphi (z)$ are respectively defined as the beam-waist diameter, beam radius and total phase factor; *z* is the propagation distance along the axis direction of the field from the beam waist located at *z* = 0 (surface 1), while *r* is the radial coordinate; *A* = (2/*π*)^1/2^ is the power normalized coefficient with *k* = 2π*n_m_*/*λ*; *f* = *πω*_0_^2^/*λ* is the Rayleigh range and φ(z) = arctan(z/f) is the Gouy phase shift. The phase shift equals *π*/4 at the position of *z* = *f*, while it approaches *π*/2 for the diffracted beam propagating to +∞. When the total reflected field of *E_r_* is coupled to the fiber, only the overlapping of the field *E_r_* with the fiber-mode field (*E_i_*) can be coupled back into the fiber, which is calculated with $\langle E_{i}|E_{d}\rangle \;=\;\int_{0}^{\infty }{E_{i}}^{*}\left(r\right)E_{d}\left(r,z\right)dr$. One can define the coupling efficiency of amplitude *η* to be:

(3) $$\eta \;=\;\left|\int_{0}^{\infty }{E_{i}}^{*}\left(r\right)E_{d}\left(r,z\right)dr\right|/{\left[\int_{0}^{\infty }E_{i}\left(r\right){E_{i}}^{*}\left(r\right)dr\int_{0}^{\infty }E_{d}\left(r,z\right){E_{d}}^{*}\left(r,z\right)dr\right]}^{1/2}$$

with ϕ(0) = 0, *z* = 2*L* and $\int_{-\infty }^{\infty }e^{-x^{2}}dx\;=\;\sqrt{\pi }$, one can finally simplify Equation (3) as follows:

(4) $$\eta \;=\;{\left[1\;+\;L^{2}\lambda^{2}/(\pi^{2}\omega_{0}^{4})\right]}^{-1/4}$$

For the sake of simplicity, one can ignore the effect of the surface imperfections on the reflectivity. From Equations (1) and (3), the normalized reflection spectrum of *I_FP_*(*λ*) can be obtained as follows:

(5) $$I_{FP(\lambda )}\;=\;R_{1}\;+\;K^{2}{\left(1\;-\;R_{1}\right)}^{2}R_{2}\;+\;2{\sqrt{R_{1}R}}_{2}\;\times \;K(1\;-\;R_{1})\cos\left[4\pi n_{m}L/\lambda \;-\;\varphi (2L)\;+\;\delta \pi \;-\;\gamma \pi \right]$$

where *ϕ*(2*L*) = arctan(2*L*/*f*) is the Gouy phase shift [49]; *K* = (1−*A*_1_)*η* e^−2*αL*^ is the total loss factor of the sensor head. From Equation (5), one can see that the interference spectrum is a cosine signal, which is generated by two-beam interference. When the phase of $4\pi Ln_{m}/\lambda \;-\;\varphi (2L)\;+\;\delta \pi \;-\;\gamma \pi$ meets with the following condition:

(6) $$4\pi n_{m}L/\lambda_{\max}\;-\;\varphi (2L)\;+\;\delta \pi \;-\;\gamma \pi \;=\;2m\pi ,\;4\pi n_{m}L/\lambda_{m\mathrm{in}}\;-\;\varphi (2L)\;+\;\delta \pi \;-\;\gamma \pi \;=\;(2m\;+\;1)\pi$$

where *m* is the order of the dip of the interference pattern, *I_FP_*(*λ*) achieves the maximum and minimum values, respectively. It reveals that the dip wavelength *λ_min_* of the interference pattern is proportional to the refractive index *n_m_* and the length *L* of the F-P cavity. Therefore, the measurement of temperature (based on the thermo-optic effect of the cavity medium) or small displacement can be realized by measuring the wavelength shift of the extremum in the interference spectrum of the F-P cavity sensor after simple derivations of Equation (6). When the length of the F-P cavity is changed in the range of less than 10 μm, the calculated variation of the Gouy phase shift *ϕ*(z) is smaller than 0.018*π*, and thus can be neglected. Due to the wavelengths of the two adjacent dips of *λ_min_*_1_ and *λ_min_*_2_ in the interference fringe, one can obtain the value of *L* from:

(7) $$L\;=\;\frac{\lambda_{\min1}\lambda_{\min2}}{2n_{m}(\lambda_{\min1}-\lambda_{\min2})},\lambda_{\min1}\;>\;\lambda_{\min2}.$$

From Equation (5), the contrast for the interference fringes can be obtained from:

(8) $$C\;=\;10log_{10}\left[\frac{I_{FP(\lambda_{\max})}}{I_{FP(\lambda_{\min})}}\right]\;=\;20log_{10}\left[\left|\frac{\sqrt{R_{1}}\;+\;K(1\;-\;R_{1})\left|\left(n_{x}\;-\;n_{m}\right)\right|/(n_{x}\;+\;n_{m})}{\sqrt{R_{1}}\;-\;K(1\;-\;R_{1})\left|\left(n_{x}\;-\;n_{m}\right)\right|/(n_{x}\;+\;n_{m})}\right|\right]$$

where $K\;=\;(1\;-\;A_{1}){\left(1\;+\;L^{2}\lambda^{2}/\pi^{2}{\omega_{0}}^{4}\right)}^{-1/4}e^{-2\alpha L}$ is the total loss factor of the sensor head, which can be obtained from the calibration of the sensor. It can be easily seen that the value of *K* can affect the fringe contrast significantly. Therefore, in order to obtain a high contrast of interference fringes, the sensor head should consist of materials with low transmission loss and a small absorption coefficient. Considering the beam waist radius *ω*_0_ of Corning SMF-28 fibers of 9.2 μm and the light wavelength *λ* = 1.55 μm, one can calculate the coupling efficiency *η* for the value of *L* = 100 μm to be η ≈ 0.864. For *L* < 100 μm, the value of the coupling efficiency *η* ranges from 0.864 to 1, meaning the loss factor caused by the beam divergence has little influence on the fringe contrast, and thus can be neglected. When *η* is less than 0.5 for the value of *L* greater than 297 μm, the loss factor will significantly affect the fringe contrast.

Hence, based on Equation (8), one can conclude that the refractive index *n_x_* of the sample can be measured from the fringe contrast measurement, which is basically independent of the temperature variation.

### 2.2. Experimental Setup

**Figure 2:**
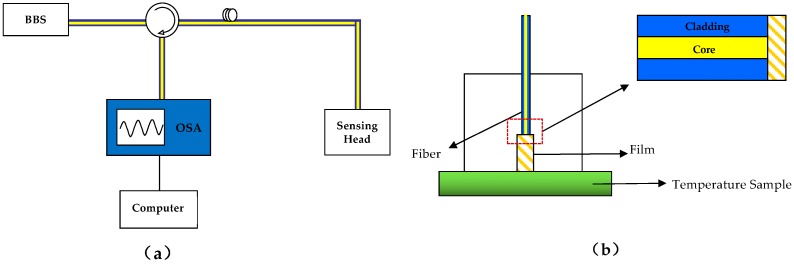
(**a**) The experimental setup of the proposed fiber-optic sensor; (**b**) Schematic of the sensing head for temperature measurement.

The typical experimental setup of the proposed sensor is shown in Figure 2a. A flattened broadband source (BBS) generates a light beam, which is launched into the sensor head via a three-port circulator. The power of BBS is about 10 mW and the bandwidth is 40 nm (1525 to 1565 nm). Then the interference spectrum reflected from the sensor head is detected by an optical spectrum analyzer (OSA), the spectral resolution of which is 0.02 nm. With a numerical aperture of 0.14 and a core diameter of 8.1 μm, Corning SMF-28 fibers are used. The fiber end of the sensing head is flatly polished vertically to the fiber axis. The function of the proposed F-P interference-based fiber-optic sensor is determined by the structure of its sensing head, which will be discussed in detail in next section.

## 3. Experiment Results and Discussions of Various Sensing Applications

The applications of the proposed F-P fiber sensor are determined by the structure of the sensing head. In order to realize a simple operation and precise measurement, a special design for each function is required for the sensing head in Figure 1a and Figure 2a.

### 3.1. Simple Fiber-Optic Temperature Sensor Based on Wavelength Interrogation [48]

**Figure 3:**
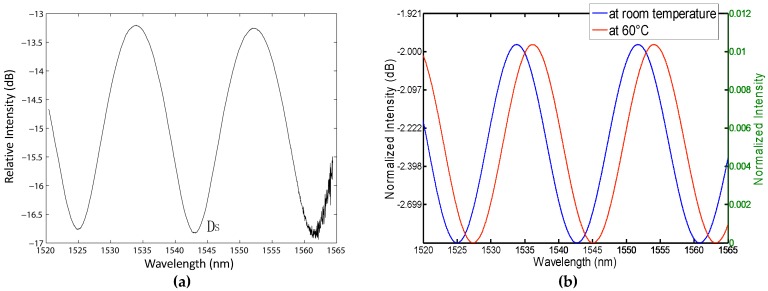
(**a**) The interference pattern received by the OSA at room temperature. Copyright (2010) Optical Engineering, from Reference [48]; (**b**) The theoretical interference spectrum at room temperature and 60 °C.

Figure 2b shows the structure of the sensing head for temperature measurement schematically. The medium for measurement is a solid film. For example, in the experiment, it was composed of the organic material SU-8 (a high-viscosity, epoxy-based photoresist), of which the RI was 1.6285 at a wavelength of 1550 nm. The head of the temperature sensor is put into the temperature test environment. As the environmental temperature changes, the thickness of the film changes because of its thermal expansion, while its RI also changes due to the thermo-optic effect. Hence, once the ambient temperature changes, from Equation (6), one can obtain expression of the dip wavelength *λ_min_* of the interference pattern of the sensor as follows:

(9) $$\Delta \lambda_{\min}/\lambda_{\min}\;=\;(dn_{film}/dT)•\Delta T/n_{film}\;+\;\alpha_{l}\Delta T$$

where *α*_l_ = (1/*L*)•(*dL*/*dT*) is the thermal expansion coefficient, and $dn_{film}/dT$ is the thermo-optic coefficient of the film. The interference pattern shifts with the temperature due to the dependence of *n_film_* and *L* of the film on the temperature. However, the thermo-optic coefficient and the thermal expansion coefficient of the fiber do not affect the wavelength shift of *λ_min_*. Therefore, the wavelength shift of *λ_min_* is only caused by the variation of the thin film. Figure 3a presents the interference spectrum of the device in air at room temperature with the intensity relative to the incident light intensity of −11.23 dBm at each wavelength. There are three dips and two peaks where the minimum and maximum occur in the interference patterns, which is in good agreement with Equation (5). Therefore, temperature sensing can be converted into the measurement of the temperature-dependent peak or dip shift of the interference spectrum.

**Figure 4:**
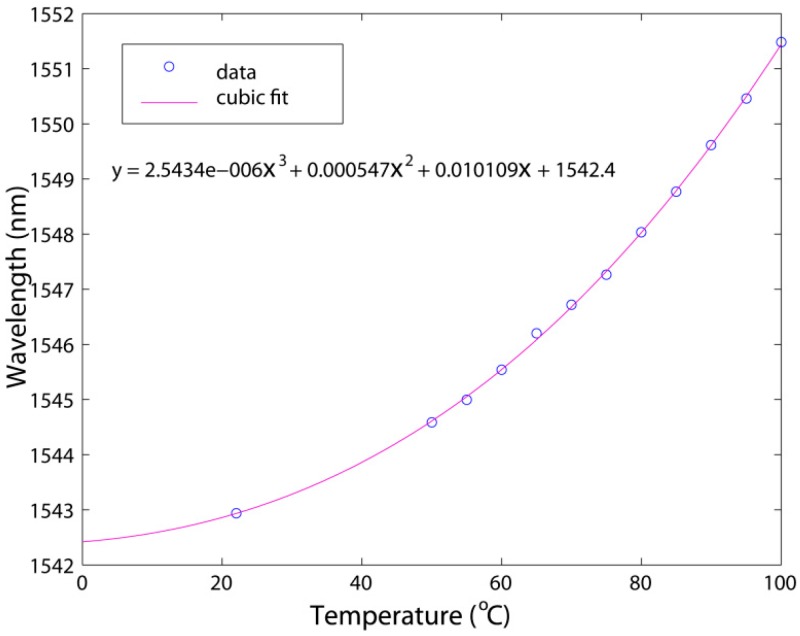
Measured wavelength shifts at different temperatures. Copyright (2010) Optical Engineering, from Reference [48].

Here a thermal controller was used to simulate the temperature change of an environment-under-test. The temperature of the thermal controller was changed from 20 to 100 °C. One of the wavelength dips (labeled as DS in Figure 3a) was chosen to detect the wavelength shift by the OSA. The measurement results indicate that the wavelength dip shifts to a longer wavelength with the increasing temperature monotonically. Figure 3b presents the theoretically normalized interference spectra in air at room temperature and 60 °C, given *n_f_* = *1.44996*, *n_film_* = 1.6285, *n_x_* = *n_air_* = *1.0003*, *L* = 40.81 μm, *K* = 0.21, based on Equation (5). One can see that the theoretical interference spectrum at room temperature agrees with the experimental one in Figure 3a. The interference peaks and dips shift to a long wavelength when the temperature changes from room temperature to 60 °C due to the change of *n_film_* and *L*. The measured wavelength shifts at different temperatures are shown in Figure 4. In the figure, the curve is a cubic fit to the experimental data, while the circles represent the experimental wavelength versus temperature. One can see that the cubic fit agrees well with the experimental data with *R*^2^ = 0.9996 [48].

One can derive an empirical formula for wavelength versus temperature from the data fitting. In addition, after the calibration described, one can use the empirical formula to calculate the ambient temperature from the measured wavelength of the chosen dip. According to the experimental results, the highest sensitivity of the thin-film sensor can reach 0.2 nm/°C, while the highest temperature resolution is 0.1 °C with the OSA resolution of 0.02 nm. The standard deviation of the temperature for one hour is less than 0.2 °C because the standard deviation of the wavelength is not more than 0.03 nm.

### 3.2. Fiber-Optic Sensors for Small Displacement Measurement [50]

**Figure 5:**
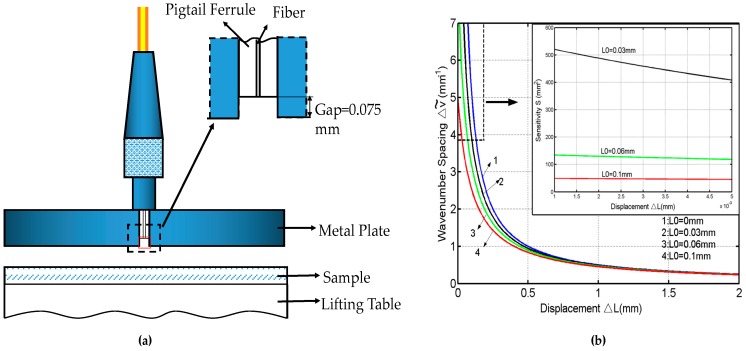
(**a**) Structure of the special design sensing head; (**b**) Simulation results with various *L*_0_. The inset: the sensitivity as a function of micro-scale displacement. Copyright (2010) Optics Communications, from Reference [50].

The structure of the special design sensing head for small displacement measurement is shown in Figure 5a. A metal plate is used to vertically clamp a PC-type (physical contact) fiber pigtail with an air gap. With this special design of the sensing head, an open F-P air cavity can be formed during the measurement. In the displacement sensing experiment, a mirror mounted on a movable table with a micrometer-positioning stage is used to simulate a measured displacement object. The medium in Figure 1a is air. Define the wavenumber $\tilde{v}$ of light to be $\tilde{v}\;=\;1/\lambda$. Therefore, from Equation (6), we can obtain the relation between the wavenumber $\tilde{v}$ and the length *L* as follows:

(10) $$2Ln_{air}\Delta \tilde{v}\;=\;1$$

where $\Delta \tilde{v}$ is the wavenumber spacing of two adjacent peaks or dips of the interference pattern. It can be seen from Equations (8) and (10) that the change of the RI of sample *n_s_* will affect the contrast and the intensities of the interference pattern but not influence the extremum positions and wavenumber spacing. The wavenumber spacing of $\Delta \tilde{v}$ is only determined by *L*. Define the initial distance between the measured object and the fiber end to be *L*_0_, and then one can obtain the displacement of Δ*L* of the measured object as follows [50]:

(11) $$\Delta L\;=\;\frac{1}{2n_{air}\Delta \tilde{v}}\;-\;L_{0}$$

One can calculate the value of Δ*L* from the measured data of $\Delta \tilde{v}$ from Equation (11). Therefore, the measurement of small displacement can be hereby achieved by monitoring the wavenumber spacing $\Delta \tilde{v}$ of the interference pattern formed by the F-P cavity.

With different initial distances of *L*_0_ (*L*_0_ = 0, 30, 60, 100 μm), an inverse relation between $\Delta \tilde{v}$ and Δ*L* is shown in Figure 5b. The measurement accuracy of Δ*L* is determined by the initial displacement of *L*_0_ and the bandwidth of the light source. To ensure that there are more than two extrema in the interference spectrum within the measurable range of a spectrum meter, the length of the gap needs to be bigger than 0.03 mm for the light source bandwidth of 1525–1565 nm, according to Equation (5). The sensitivity of *S* is defined and can be calculated from Equation (11) as follows:

(12) $$S\;=\;\left|\frac{d\Delta \tilde{v}}{d\Delta L}\right|\;=\;\frac{1}{2{\left(\Delta L\;+\;L_{0}\right)}^{2}}.$$

The relation between *S* and Δ*L* with different initial distances of *L*_0_ is shown in the inset in Figure 5b. A smaller initial distance and/or larger light source bandwidth can improve the sensitivity and then the resolution of the sensor. With the OSA resolution of 0.02 nm, the highest theoretical resolutions of displacement Δ*L* are 16 nm, 60 nm, and 166 nm for the initial distance *L*_0_ of 30 μm, 60 μm, and 100 μm, respectively. Thus, the proposed displacement sensor has a capability for achieving a high displacement resolution of 16 nm for an initial distance of 30 μm.

One can realize small displacement measurements by detecting the value of $\Delta \tilde{v}$ between two adjacent peaks or dips. In order to eliminate the measurement error for small displacement measurements caused by the change of *L*_0_, a quick and onsite *L*_0_ calibration is required before displacement measurements. It can be done from the $\Delta \tilde{v}$ measurement at no displacement and the *L*_0_ calculation based on Equation (11).

**Figure 6:**
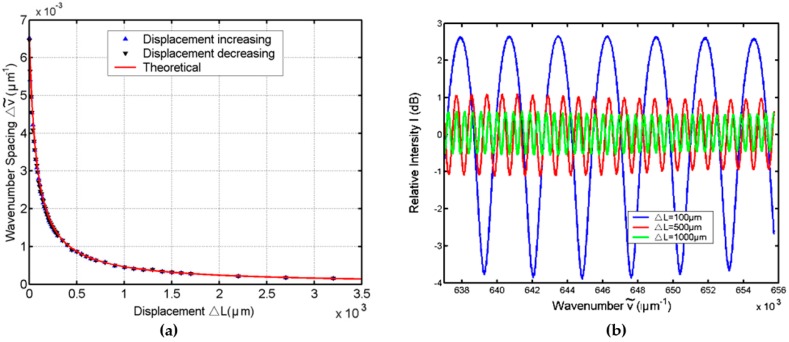
(**a**) Experimental and theoretical plots for the wavenumber spacing versus the displacement of the mirror; (**b**) The interference fringes with various displacements. Copyright (2010) Optics Communications, from Reference [50].

Figure 6a displays the measured results and theoretical values of the wavenumber spacing $\Delta \tilde{v}$ versus displacement. The solid curve represents the theoretical values with the calibrated *L*_0_ = 75 μm. From the figure, the up-triangles represent the measured $\Delta \tilde{v}$ versus displacement Δ*L* in increasing rounds, while the down-triangles represent $\Delta \tilde{v}$ versus Δ*L* in decreasing rounds. It can be seen that the experimental results are in good agreement with the theoretical curve with *R*^2^ = 0.9962, which verifies the validity of the analysis above and the system feasibility of the technique. Figure 6b presents the interference fringes with the displacements of the mirror of 100 μm, 500 μm, and 1000 μm, respectively. It can be seen that the wavenumber spacing of $\Delta \tilde{v}$ varies with the displacement, and the contrast of the fringes also decreases due to the energy loss. According to the figure, the contrast for the interference fringes is still visible when the displacement of the mirror increases to 1000 μm.

One can see from Equations (10) and (11) that the material properties of the measured object have no effect on the wavenumber spacing of $\Delta \tilde{v}$. It reveals that the technique is suitable for displacement measurements of various reflective objects with different materials. The sensor could be extended to the measurements of vibration or acceleration, and has the potential capability of remote and on-line sensing.

### 3.3. Temperature-Insensitive Sensor for RI Measurement of Liquid [51]

The structure of the sensing head for the RI measurement of a liquid is similar to that of the temperature sensor. The difference is that the sensing tip of the RI sensor is put into a liquid sample of which the RI is to be measured. The two interfaces, fiber–thin-film and thin-film–liquid, form an F-P cavity. Then, one can substitute *n_m_* = *n_film_* and *n_x_* = *n_l_* into Equation (8) to obtain the fringe contrast.

It reveals that the variation of the contrast of the interference fringe with the increase of *n_l_* is a multi-interval and non-monotonous function. *R*_1_, *K* and *n_film_* are not sensitive to temperature variation due to the small thermo-optic coefficient of the film (about1.86 × 10^−4^ K^−1^). Thus, the *n_l_*-sensing is temperature-insensitive. The equivalent total loss factor *K* of the sensor head should be calibrated from fitting the interference spectrum pattern with Equation (5) under the sensor head being put in air or a liquid with a known RI, before any RI sensing.

**Figure 7:**
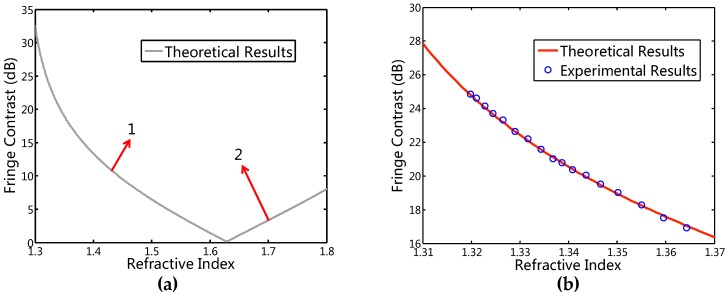
(**a**) Theoretical variation of the fringe contrast versus RI; (**b**) Measured fringe contrast versus the RI, showing the experiment results agree well with the theoretical results.

Figure 7a shows the theoretical curve of the fringe contrast versus the RI within the range from 1.3 to 1.8, which is the range of most liquids, with the parameters of *K* = 0.4972, *R*_1_ = 0.0034 and *n_film_* = 1.6285 [51]. One can see from Figure 7a that the curve can be divided into two branches, labeled as “1” and “2”, respectively, with the boundary of *n_film_* = 1.6285. The contrast of the interference fringe decreases with the increment of the RI within the range from 1.3 to 1.6285 monotonously, while it monotonously increases within the RI ranging from 1.6285 to 1.8. Thus, the RI measurement can be achieved from branch 1 or 2, as determined by *n_l_* being smaller or bigger than *n_film_*.

To validate the technique, NaNO_3_-water solutions of various concentrations were chosen for RI measurement in the previous experiment [51]. The experimental results of the fringe contrast versus the RI are shown in Figure 7b. One can see that the experimental results agree well with the theoretical results in branch 1 (Figure 7a) as based on Equation (8). With the relative intensity resolution of the OSA of 0.001 dB, the resolution of the proposed sensor is ~5 × 10^−6^ for the RI ranging from 1.314 to 1.365, based on the average RI sensitivity of 205 dB/RI in Figure 7b. Besides the temperature-independent measurement, the sensor has a potential for measuring the environment temperature and the RI of liquid simultaneously.

### 3.4. Fiber Optic F-P Interferometric Sensor for RI Measurement of Solid [52]

The structure of the sensing head of the fiber-optic sensor for the RI measurement of a solid is very similar to the sensing head for the small displacement measurement mentioned above. The difference is that the metal plate needs to touch the solid-under-test tightly during the RI measurement, and then a constant F-P air cavity is formed. Additionally, the surfaces of the measured sample and the fiber end are able to stay parallel, and the sensing fiber end can be protected from being damaged. It gives the parameters of *n_m_* = *n_air_* = *1.0003*, *δ* = 0 and *γ* = 1, because there is a π-phase shift on surface 2 for when the light reflects from an optically denser medium. Define *n_s_* to be the RI of the solid-under-test. Then, with *n_m_* = *n_air_* and *n_x_* = *n_s_*, the fringe contrast of the interference spectrum can be obtained from Equation (8).

To calculate the total loss factor *K*, a quick *C*_0_ calibration with a sample of the known RI is required. That is, before the measuring, the fringe contrast *C*_0_ of a sample with a known RI (such as silicon dioxide) should be measured as reference data after the whole sensing system is set up, without any modification of the sensing system. With *n_m_* = *n_air_* = *1.0003, n_f_* = *1.44996*, one can calculate the value of *K* easily through the *C*_0_ calibration. After the calibration, the RI of any solid sample can be measured by using the fringe contrast of its interference spectrum with this sensor.

**Figure 8:**
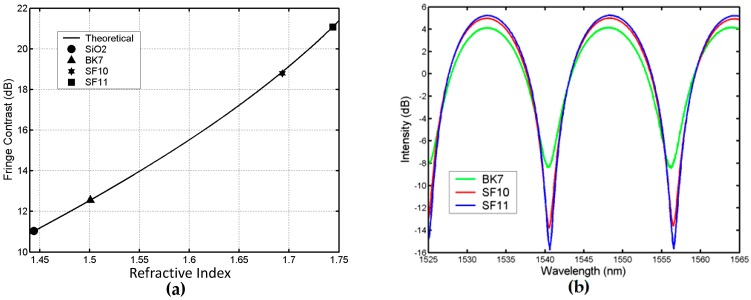
(**a**) Theoretical variation of the fringe contrast versus RI and experimental fringe contrasts of the measured samples; (**b**) Reflection spectra of the sensor for measured samples of BK7, SF10, and SF11, respectively. Copyright (2010) Applied Optics, from Reference [52].

In the experiment [52], the length of the gap is 0.075 mm. To ensure that there are not less than two extrema in the interference spectrum in the measurable range of a spectrum meter, the length of the gap ought to be bigger than 0.03 mm for a light source bandwidth of 1525–1565 nm, according to Equation (6). We chose silicon dioxide (SiO_2_) glass with a RI of 1.443 at a wavelength of 1529.52 nm as the calibration sample [52]. The sample should be cleaned before measurement as the sample surface is required to be planar and smooth, without contamination. The interference fringe of measuring SiO_2_ received by OSA is similar to the one in Figure 3a, and the fringe contrast of the interference spectrum is 11.033 dB. From Equation (8), the total loss factor of *K* was calculated to be about 0.5868 for the sensing head used in the setup. With the maximum deviation of 1 °C, the ambient temperature was controlled at 20 °C. Three styles of optical glasses of BK7, SF10, and SF11 were used as test samples for RI measurement. Figure 8b shows the reflection spectra of the sensor for BK7, SF10, and SF11, respectively.

From Figure 8b, the contrasts of the interference fringes of BK7, SF10, and SF11 can be obtained respectively as 12.560 dB, 18.796 dB, and 21.052 dB. Therefore, with *n_m_* = *n_air_* and *n_x_* = *n_s_*, one can use Equation (8) to calculate the reflective indices of BK7, SF10, and SF11. It gives *n_BK_*_7_ = 1.501*, n_SF_*_10_ = 1.692 and *n_SF_*_11_ = 1.743, respectively [52], which are very close to the values of *n_BK_*_7_ = 1.5009*, n_SF_*_10_ = 1.6931 and *n_SF_*_11_ = 1.7438, respectively, at a wavelength of 1529.6 nm and a temperature of 20 °C (193.15 K) in SCHOTT Optical Glass Data Sheets (2015) [53]. The deviations between the experimental results and SCHOTT data are about 0.0001, 0.001, and 0.001, respectively. With Equation (8) and the value of *K* = 0.5868, Figure 8a presents the theoretical curve of the fringe contrast versus the RI and the measured fringe contrasts of the given four samples (including SiO_2_) versus the refractive indices from the SCHOTT data. One can see that the experimental results agree well with the theoretical values, which verifies the feasibility of the technique and the veracity of the analysis above. According to Figure 8a, the fringe contrast increases with the increase of the RI, showing the relation between them as nearly a linear function with a slope of $dC/dn_{s}\;\approx \;33dB/RI$. With the power resolution of an OSA of 0.01 dB or 0.001 dB, the resolution for the glass RI measurement is about 3 × 10^−4^ or 3 × 10^−5^.

## 4. Conclusions and Outlook

The fiber-end F-P interferometric fiber-optic sensors for measurements of various parameters have been reviewed. We firstly give a general model for the basic operation principle of F-P interferometric optical fiber sensors by including the analysis of the Gouy effect and the beam divergence in an F-P cavity. It is theoretically and experimentally shown that temperature and small displacement sensing can be realized by measuring the interference peak or dip shift in the F-P interference spectrum, and the fringe contrast of the interference spectrum can be used for measuring the RI of liquids or solids. The temperature sensor can achieve the highest resolution of ~0.1 °C within the measured temperature range from 20 to 100 °C, while the liquid RI sensor can offer the RI resolution of 5 × 10^−6^. The proposed displacement sensing system could achieve a high resolution measurement of 16 nm with the initial distance of 30 μm.

The fiber-end F-P interferometric optic sensors have advantages such as compact size, fast response, and high sensitivity, which are highly desired for many sensing applications. They can be easily extended to other functions and applications of interferometric fiber-optic sensors.

As an outlook, our future work may be focused on (1) utilizing the sensors to operate in extreme environments, such as temperatures over 1000 °C, or high-pressure or corrosive environments; (2) expanding the F-P interferometric fiber-optic sensor to simultaneous multi-parameter measurements; (3) expanding the applications to chemical sensors and biosensors, such as the detection of toxic and biological threats; (4) expanding the applications of the medical field by combining the sensor with medical techniques, such as measuring hematocrit levels in human blood or the intra-tissue pressure measurements of human organs.
